# Supplementary material for: Angiogenin mediates paternal inflammation-induced metabolic disorders in offspring through sperm tsRNAs
Source: Nat Commun. 2021 Nov 29;12:6673. doi: 10.1038/s41467-021-26909-1 (PMC8630171; doi:10.1038/s41467-021-26909-1)
Supplement: Supplementary file 1 — Supplementary Information [file 41467_2021_26909_MOESM1_ESM.pdf]

Supplementary Information

Angiogenin mediates paternal inflammation-induced metabolic disorders in offspring through sperm tsRNAs

Yanwen Zhang, Li Ren, Xiaoxiao Sun, Zhilong Zhang, Jie Liu, Yining Xin, Jianmin Yu,

Yimin Jia, Jinghao Sheng, Guo-fu Hu, Ruqian Zhao, Bin He*

*Corresponding author

E-mail: heb@njau.edu.cn

This PDF file includes:

Supplementary Figs. 1 to 7

Supplementary Tabs. 1 to 7

Supplementary Fig. 1

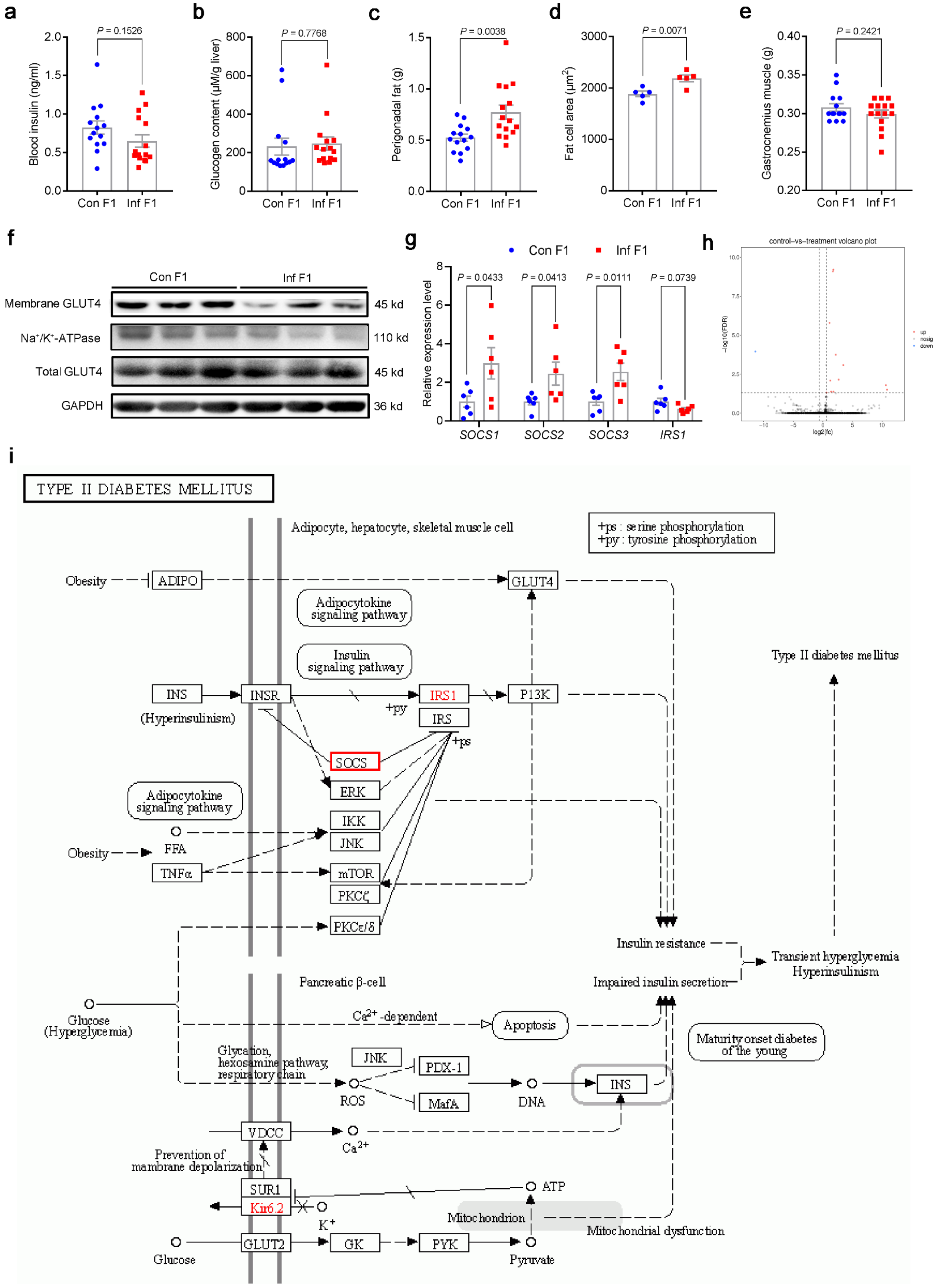

Supplementary Fig. 1 Metabolic parameters and expression of GLUT4 in F1 males with paternal inflammation.

a Blood insulin concentration in Con F1 and Inf F1male mice at 15 weeks of age. **b** The content of hepatic glycogen in Con F1 and Inf F1 male mice at 15 weeks of age. **c** The perigonadal fat mass in Con F1 and Inf F1male mice at 15 weeks of age. **d** The fat cell area in Con F1 and Inf F1male mice at 15 weeks of age. n = 5 mice per group. **e** The gastrocnemius muscle mass in Con F1 and Inf F1male mice at 15 weeks of age. In **a-c** and **e**, n = 14 in Con and n = 15 in inf. **f** Western blots of total (GAPDH was used as the loading control) and plasma membrane (Na^+/K^+ ATPase was used as the loading control) GLUT4 content in the gastrocnemius muscle isolated from Inf F1 and Con F1 mice at 15 weeks of age. n = 6 mice per group. **g** The relative expression levels of *SOCS1, SOCS2, SOCS3* and *ISR1* mRNA in the gastrocnemius muscle of Inf F1 and Con F1 male mice. n = 6 mice per group. Statistical analysis was performed by two-tailed unpaired Student’s t-test (**a**-**e**, **g**). All data are plotted as means ± SEM, each dot represents one mouse. **h** Volcano plots visualize differential gene expression in gastrocnemius muscle of Inf F1 and Con F1 male mice. Red and blue points are differentially expressed transcripts, having both a significant test statistic as well as a log2 fold change of 2 or greater. **i** Type II diabetes mellitus pathway in gastrocnemius muscle of Inf F1 and Con F1 male mice. Genes with red box indicate differentially regulated genes in Inf F1 male mice vs. Con F1 male mice ($P < 0.05$). Source data are provided as a Source Data file.

Supplementary Fig. 2

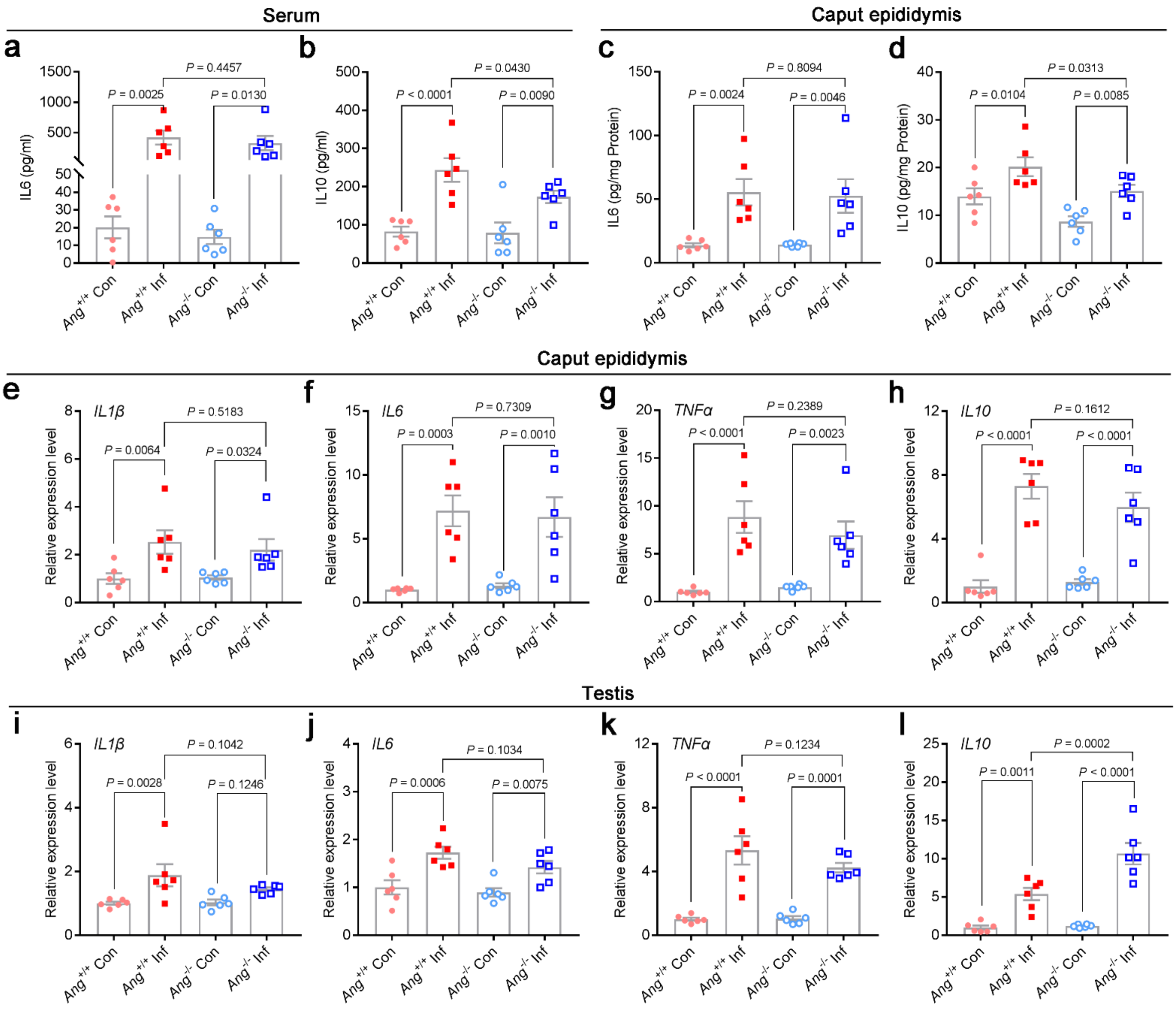

Supplementary Fig. 2 The content of inflammatory cytokines in serum, caput epididymis and testis of *Ang*$^{+/+}$ and *Ang*$^{-/-}$ mice with or without LPS treatment.

Male *Ang*$^{+/+}$ and *Ang*$^{-/-}$ mice were treated with LPS (Inf) or saline (Con) to establish the inflammatory model. **a**-b ELISA of IL6 (**a**) and IL10 (**b**) in the serum 12 h after LPS and saline treatment. **c**-**d** ELISA of IL6 (**c**) and IL10 (**d**) in the caput epididymis 12 h after LPS and saline treatment. **e**-**h** The relative expression levels of *IL1β* (**e**), *IL6* (**f**), *TNFα* (**g**) and *IL10* (**h**) mRNA in the caput epididymis 24 h after LPS or saline treatment. **i**-**l** The relative expression levels of *IL1β* (**i**), *IL6* (**j**), *TNFα* (**k**) and *IL10* (**l**) mRNA in the testis 24 h after LPS or saline treatment. Statistical analysis was performed by two-tailed, one-way analysis of variance (ANOVA), uncorrected Fisher's least significant difference (LSD). n = 6 mice per group. All data are plotted as means ± SEM, each dot represents one mouse. Source data are provided as a Source Data file.

Supplementary Fig. 3

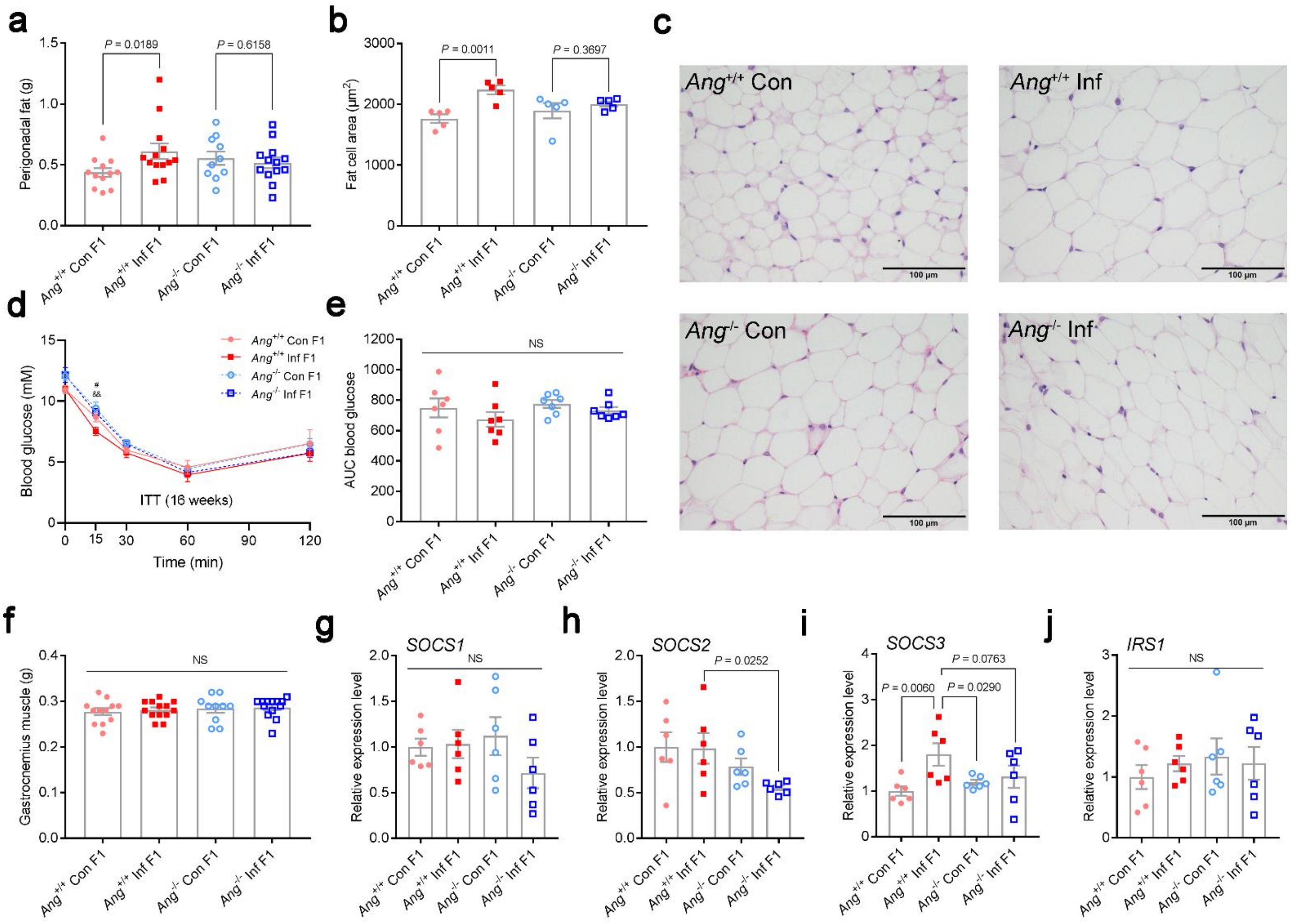

Supplementary Fig. 3 Metabolic parameters of F1 males from inflammatory and *Ang*-deleted mice.

a The perigonadal fat mass of F1 males at 16 weeks of age. **b** The fat cell area of F1 males at 16 weeks of age. n = 5 mice per group. **c** Representative images showing adipocyte area in perigonadal fat of F1 males in each group. n = 5 mice per group. Scale bar = 100 μm. **d** Blood glucose levels as assayed by ITT of F1 male mice at 15 weeks of age. $^{\&\&}P < 0.01$ ($Ang^{+/+}$ Inf versus $Ang^{-/-}$Con); $^{\#}$ $P < 0.05$ ($Ang^{+/+}$ Inf versus $Ang^{-/-}$ Inf). n = 7 mice per group. **e** AUC statistics for **d**. n = 7 mice per group. **f** The gastrocnemius muscle mass of F1 males at 16 weeks of age. In **a** and **f**, n = 12 in $Ang^{+/+}$ Con group, n = 13 in $Ang^{+/+}$ Inf group, n = 10 in $Ang^{-/-}$ Con group, and n = 13 in $Ang^{-/-}$ Inf group. **g**-**j** The relative expression levels of *SOCS1, SOCS2, SOCS3* and *ISR1* mRNA in the gastrocnemius muscle of F1 males. n = 6 mice per group. Statistical analysis was performed by two-tailed, one-way ANOVA (**a-b**, **e-j**) or two-way ANOVA (**d**), uncorrected Fisher's LSD. All data are plotted as means ± SEM, each dot represents one mouse. NS, not significant. Source data are provided as a Source Data file.

Supplementary Fig. 4

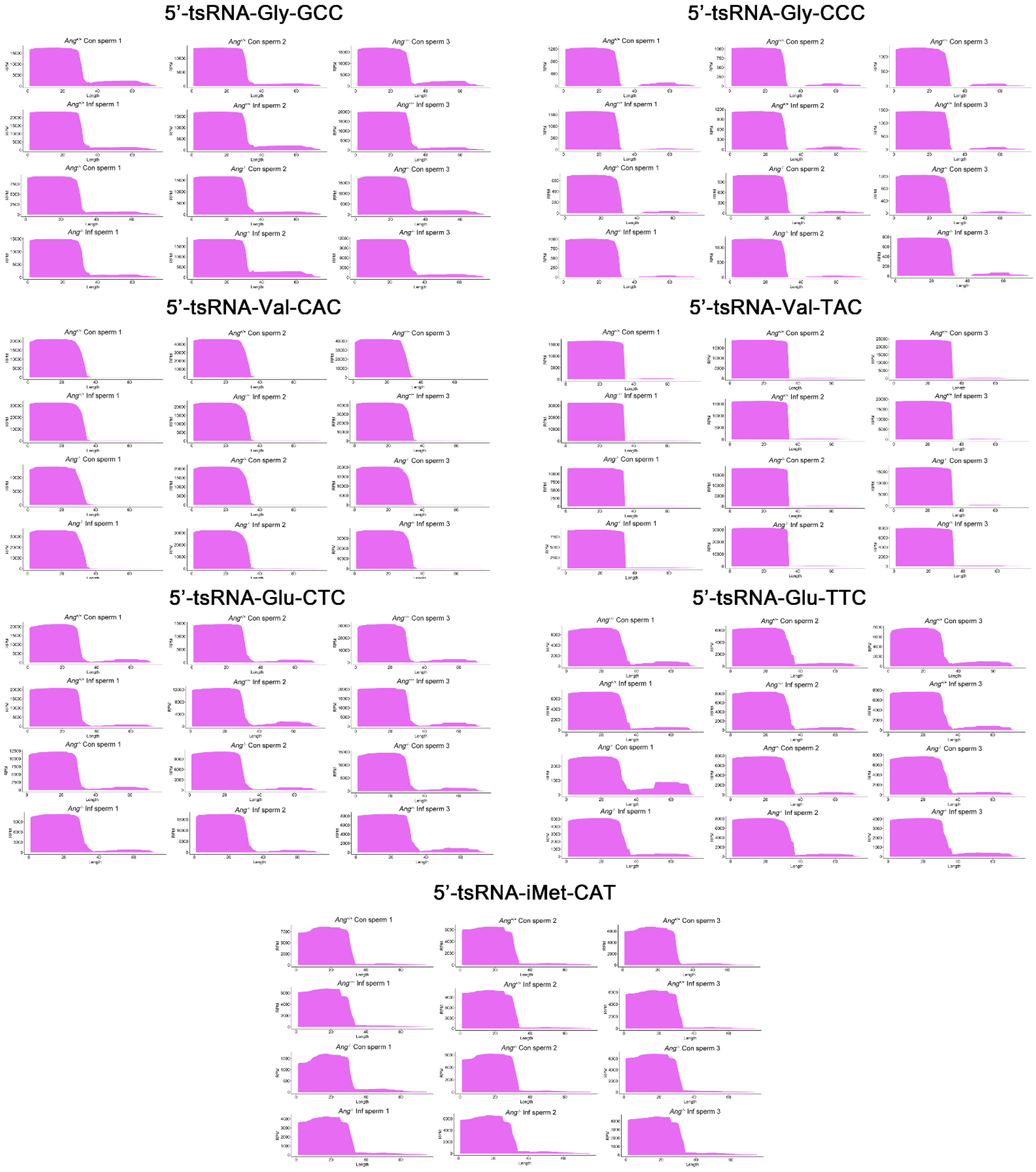

Supplementary Fig. 4 Loci mapping information of tsRNAs in different tRNAs for *Ang*$^{+/+}$ Con, *Ang* $^{+/+}$ Inf, *Ang*$^{-/-}$ Con, and *Ang*$^{-/-}$ Inf sperm.

RPM: reads per million.

Supplementary Fig. 5

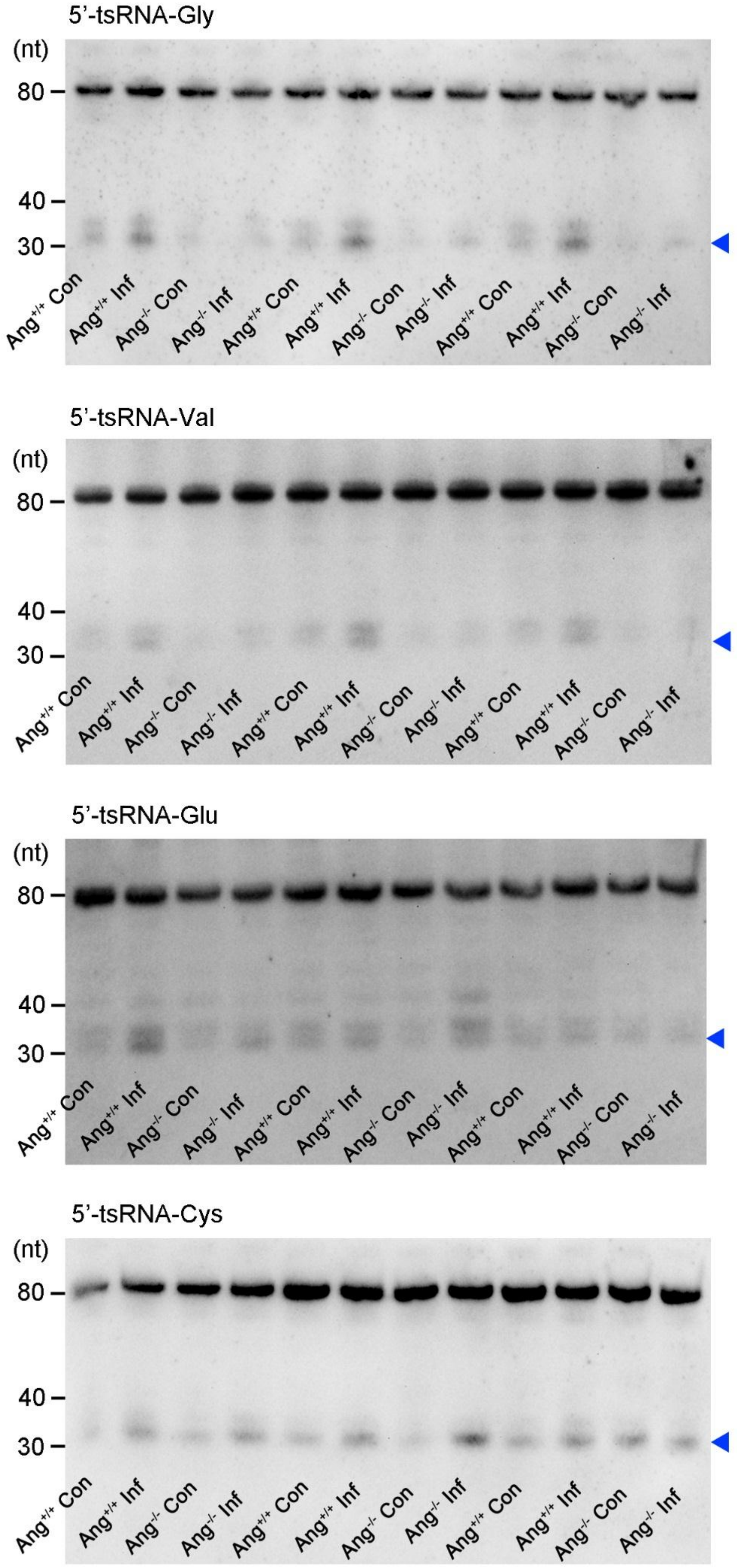

Supplementary Fig. 5 Expression of tsRNAs in the caput epididymis from *Ang*$^{+/+}$ Con, *Ang*$^{+/+}$ Inf, *Ang*$^{–/–}$ Con, and *Ang*$^{–/–}$ mice.

Northern blot analyses of 5′tsRNA-Gly, 5′tsRNA-Val, 5′tsRNA-Glu, and 5′tsRNA-Cys (shown by arrow heads) in the caput epididymis. Total RNAs from the caput epididymis were run on a 15% denatured PAGE gel as shown as a loading control. n = 3 mice per group.

Supplementary Fig. 6

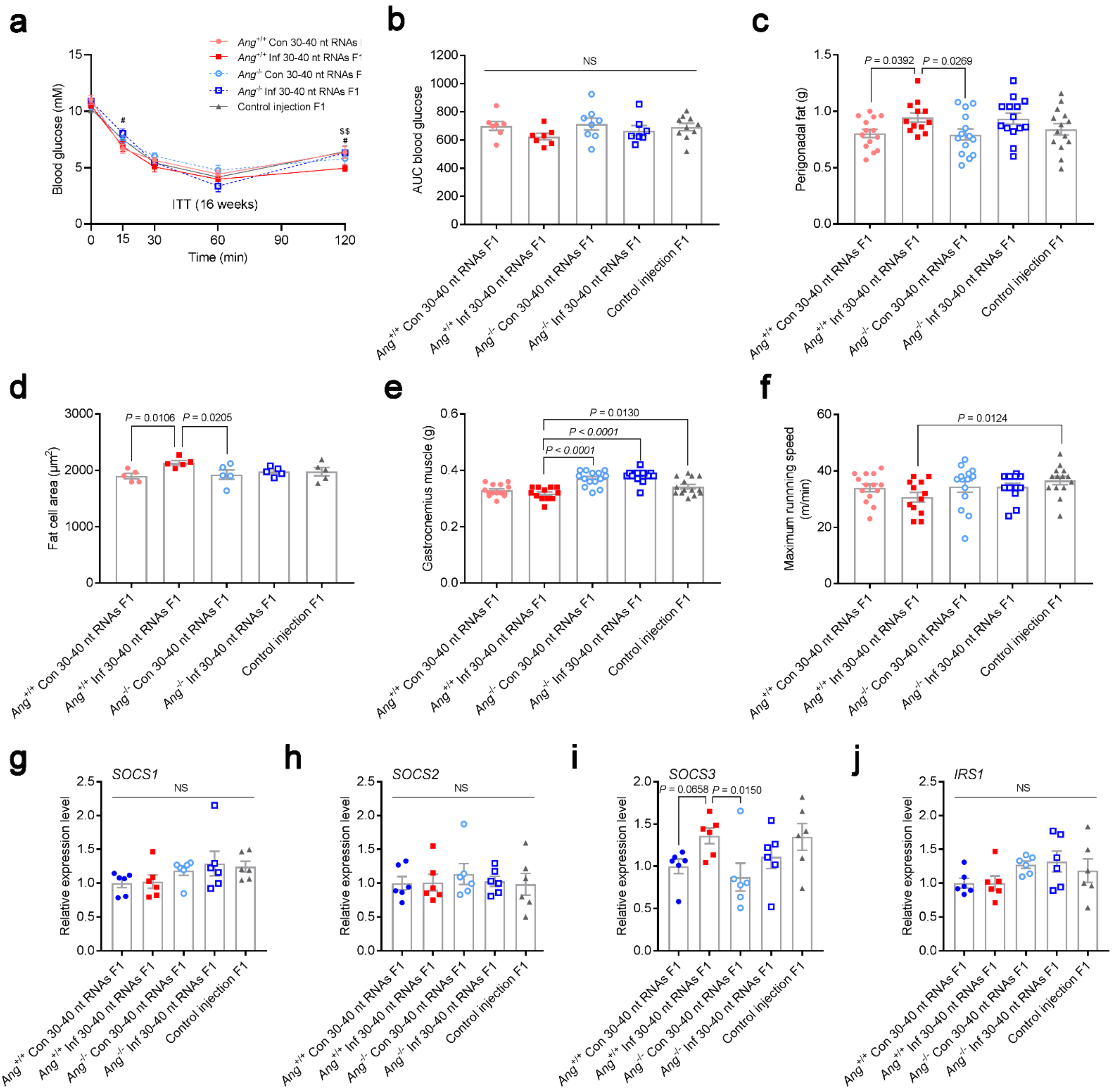

Supplementary Fig. 6 Metabolic parameters of F1 males generated by zygotic injection of sperm 30-40 nt RNAs.

a Blood glucose during ITT of F1 males generated from sperm 30-40 nt RNAs injection at 16 weeks of age. Statistical analysis was performed by two-tailed, two-way ANOVA, uncorrected Fisher's LSD. $^{\#}$ $P < 0.05$ (*Ang*$^{+/+}$ Inf versus *Ang*$^{-/-}$ Inf); $^{\$\$}$ $P < 0.01$ (*Ang*$^{+/+}$ Inf versus control injection). n = 7 mice per group. **b** AUC statistics for **a**. n = 7 mice per group. **c** The perigonadal fat mass of F1 males at 17 weeks of age. **d** The fat cell area of F1 males at 17 weeks of age. n = 5 mice per group. **e** The gastrocnemius muscle mass of F1 males at 17 weeks of age. **f** Treadmill test results of F1 males at 12 weeks of age. In **c** and **e**-**f**, n = 14 in *Ang*$^{+/+}$ Con group, n = 12 in *Ang*$^{+/+}$ Inf group, n = 14 in *Ang*$^{-/-}$ Con group, n = 14 in *Ang*$^{-/-}$ Inf group, and n = 14 in control group. **g**-**j** The relative expression levels of *SOCS1* (**g**), *SOCS2* (**h**), *SOCS3* (**i**) and *ISR1* (**j**) mRNA in the gastrocnemius muscle of F1 males. n = 6 mice per group. In **b-j**, statistical analysis was performed by two-tailed, one-way ANOVA, uncorrected Fisher's LSD. Source data are provided as a Source Data file.

Supplementary Fig. 7

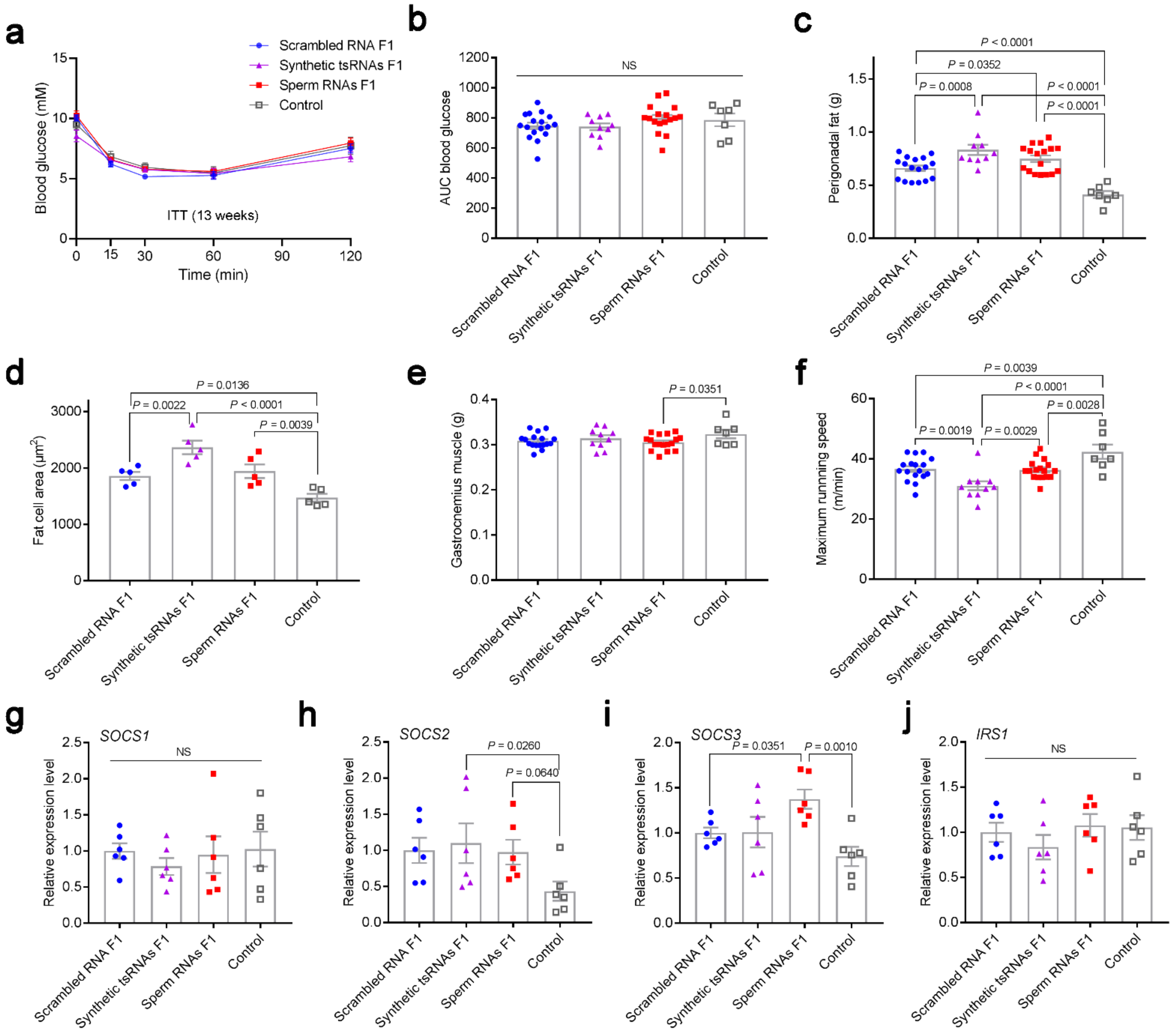

Supplementary Fig. 7 Metabolic parameters of F1 males generated by zygotic injection of sperm 30-40 nt RNAs or synthetic tsRNAs.

a Blood glucose during ITT of F1 males generated from scrambled RNA, synthetic tsRNAs, sperm 30-40 nt RNAs from *Ang*$^{+/+}$ Inf mice (Sperm RNAs) injection and control mice at 13 weeks of age. Statistical analysis was performed by two-tailed, two-way ANOVA, uncorrected Fisher's LSD. **b** AUC statistics for **a**. **c** The perigonadal fat mass of F1 males at 15 weeks of age. **d** The fat cell area of F1 males at 15 weeks of age. n = 5 mice per group. **e** The gastrocnemius muscle mass of F1 males at 15 weeks of age. **f** Treadmill test results of F1 males at 11 weeks of age. In **a**-**c** and **e**-**f**, n = 17 in scrambled RNA group, n = 10 in synthetic tsRNAs group, n = 17 in sperm RNAs, and n = 7 in control group. **g**-**j** The relative expression levels of *SOCS1* (**g**), *SOCS2* (**h**), *SOCS3* (**i**) and *ISR1* (**j**) mRNA in the gastrocnemius muscle of F1 males. n = 6 mice per group. In **b-j**, statistical analysis was performed by two-tailed, one-way ANOVA, uncorrected Fisher's LSD. All data are plotted as means ± SEM, each dot represents one mouse. NS, not significant. Source data are provided as a Source Data file.

Supplementary Tab. 1 Summary of reproductive data from Con and Inf mice.

	Con	Inf
Number of F0 males	6	6
Number of F0 females	12	12
Number of pregnant	7	6
Fertility index (%)	58.3	50.0
Total number of pups born	44	41
Mean live pups/litter	6.29±2.66	6.83±1.95
Sex ratio (male/female)	25/19	23/18

Supplementary Tab. 2 Summary of reproductive data from *Ang*$^{+/+}$ Con, *Ang*$^{+/+}$ Inf, *Ang*$^{-/-}$ Con, and *Ang*$^{-/-}$ Inf mice.

	Ang$^{+/+}$ Con	*Ang*$^{+/+}$ Inf	*Ang*$^{-/-}$ Con	*Ang*$^{-/-}$ Inf
Number of F0 males	6	6	6	6
Number of F0 females	12	12	12	12
Number of pregnant	6	5	7	6
Fertility index (%)	50.0	41.7	58.3	50.0
Total number of pups born	45	39	41	41
Mean live pups/litter	7.50±1.61	7.80±1.17	5.86±2.03	6.83±1.67
Sex ratio (male/female)	20/25	19/20	21/20	24/17

Supplementary Tab. 3 Summary of outcome after sperm 30-40 nt RNAs and control injection into normal zygotes.

	Injected zygotes	Transferred zygotes	Live born (% transfer)
Ang$^{+/+}$ Con	100	100	25 (25%)
Ang$^{+/+}$ Inf	100	100	36 (36%)
Ang$^{-/-}$ Con	100	100	40 (40%)
Ang$^{-/-}$ Inf	100	100	47 (47%)
Control injection	100	100	35 (35%)

Note: For embryo transfer, zygotes (10-15) were transferred into one side of oviduct, both oviducts get transfers, with an amount of 25 zygotes transferred each surrogate mother. Four mice were used as surrogate mother every groups.

Supplementary Tab. 4 Sequences of synthetic tsRNAs and scrambled RNA injected into normal zygotes.

tsRNA	Sequences (5' to 3')
tsRNA-Glu-CTC; tsRNA-Glu-TTC	UCCCUGGUGGUCUAGUGGUUAGGAUUCGGCG
tsRNA-Glu-TTC	UCCCACAUGGUCUAGCGGUUAGGAUUCCUGGUU
tsRNA-Gly-CCC	GCGCCGCUGGUGUAGUGGUAUCAUGCAAGAUUC
tsRNA-Gly-GCC	GCAUUUGUGGUUCAGUGGUAGAAUUCUCGCC
tsRNA-Gly-GCC; tsRNA-Gly-CCC	GCAUUGGUGGUUCAGUGGUAGAAUUCUCGCC
tsRNA-Gly-GCC; tsRNA-Gly-CCC	GCAUUGGUGGUUCAGUGGUAGAAUUCUCGC
tsRNA-iMet-CAT	AGCAGAGUGGCGCAGCGGAAGCGUGCUGGGCCC
tsRNA-Val-CAC; tsRNA-Val-AAC	GUUUCCGUAGUGUAGUGGUUAUCACGUUCGCC
tsRNA-Val-TAC	GGUUCCAUAGUGUAGCGGUUAUCACGUCUGCUUU
tsRNA-Val-TAC	GGUUCCAUAGUGUAGCGGUUAUCACGUCUGCUU
Scrambled RNA	CCUCCCAAAGUGCUGGGAUUACAGGCGUGAG

Supplementary Tab. 5 Summary of outcome after scrambled RNA, synthetic tsRNAs and sperm 30-40 nt RNAs from *Ang*$^{+/+}$ Inf mice injection into normal zygotes.

	Injected zygotes	Transferred zygotes	Live born (% transfer)
Scrambled RNAs	100	100	47 (47%)
Synthetic tsRNAs	100	100	17 (17%)
Sperm 30-40 nt RNAs	100	100	47 (47%)

Note: For embryo transfer, zygotes (10-15) were transferred into one side of oviduct, both oviducts get transfers, with an amount of 25 zygotes transferred each surrogate mother. Four mice were used as surrogate mother every groups.

Supplementary Tab. 6 Nucleotide sequences of specific primers for qRT-PCR.

Target genes	Sequences (5’ to 3’)	GenBank No.
SOCS1	F: CGAGACCTTCGACTGCCTTT<br>R: AGTCACGGAGTACCGGGTTA	NM_001271603.1
SOCS2	F: CAGCTGGACCGACTAACCTG<br>R: TGAACAGTCCCATTCCGTGG	NM _007706.4
SOCS3	F: ACCCTCCAGCATCTTTGTCG<br>R: GAACTCCCGAATGGGTCCAG	NM_007707.3
IRS1	F: GACATTGGAGGTGGGTCCAG<br>R: TTACCGCCACCACTCTCAAC	NM_010570.4
Ang	F: TCTGGCTCAGGATGACTCCA<br>R: GACCTGGAAGGGAGACTTGC	NM_007447.3
IL1β	F: CTTCAGGCAGGCAGTATC<br>R: CAGCAGGTTATCATCATCATC	NM_008361.4
TNFα	F: CCTATGTCTCAGCCTCTTCTCA<br>R: CTTCTCATCCCTTTGGGGAC	NM_001278601.1
IL6	F: GCCTTCTTGGGACTGATGCT<br>R: GACAGGTCTGTTGGGAGTGG	NM_031168.2
IL10	F: CAGAGAAGCATGGCCCAGAA<br>R: GCTCCACTGCCTTGCTCTTA	NM_010548.2
GAPDH	F: TCTCCTGCGACTTCAACA<br>R: TGTAGCCGTATTCATTGTCA	GU214026.1

Supplementary Tab. 7 Nucleotide sequences of specific probes for Northern blot.

Northern blot probe	Sequences (5’ to 3’)
tsRNA-Gly	TCTACCACTGAACCACCAAT
tsRNA-Glu	CCACTAGACCACCAGGGA
tsRNA-Val	CCACTACACTACGGAAAC
tsRNA-Cys	CCACTGAGCTATACCCCC
