# Supplementary material for: Angiogenin mediates paternal inflammation-induced metabolic disorders in offspring through sperm tsRNAs
Source: Nat Commun. 2021 Nov 29;12:6673. doi: 10.1038/s41467-021-26909-1 (PMC8630171; doi:10.1038/s41467-021-26909-1)
Supplement: Supplementary file 3 — Description of Additional Supplementary Files [file 41467_2021_26909_MOESM3_ESM.pdf]

Description of Additional Supplementary Files

Title: Supplementary Data 1
Description: Abundance of sperm 5'-tsRNAs in inflammatory and *Ang*-deleted mice
