# Supplementary material for: Angiogenin mediates paternal inflammation-induced metabolic disorders in offspring through sperm tsRNAs
Source: Nat Commun. 2021 Nov 29;12:6673. doi: 10.1038/s41467-021-26909-1 (PMC8630171; doi:10.1038/s41467-021-26909-1)
Supplement: Supplementary file 5 — Reporting Summary [file 41467_2021_26909_MOESM5_ESM.pdf]

nature portfolio

Corresponding author(s): Bin He

Last updated by author(s): Sep 7, 2021

Reporting Summary

Nature Portfolio wishes to improve the reproducibility of the work that we publish. This form provides structure for consistency and transparency in reporting. For further information on Nature Portfolio policies, see our Editorial Policies and the Editorial Policy Checklist.

Statistics

For all statistical analyses, confirm that the following items are present in the figure legend, table legend, main text, or Methods section.

n/a	Confirmed	
☐	☒	The exact sample size (*n*) for each experimental group/condition, given as a discrete number and unit of measurement
☐	☒	A statement on whether measurements were taken from distinct samples or whether the same sample was measured repeatedly
☐	☒	The statistical test(s) used AND whether they are one- or two-sided<br>*Only common tests should be described solely by name; describe more complex techniques in the Methods section.*
☐	☒	A description of all covariates tested
☒	☐	A description of any assumptions or corrections, such as tests of normality and adjustment for multiple comparisons
☐	☒	A full description of the statistical parameters including central tendency (e.g. means) or other basic estimates (e.g. regression coefficient) AND variation (e.g. standard deviation) or associated estimates of uncertainty (e.g. confidence intervals)
☐	☒	For null hypothesis testing, the test statistic (e.g. *F*, *t*, *r*) with confidence intervals, effect sizes, degrees of freedom and *P* value noted<br>*Give P values as exact values whenever suitable.*
☒	☐	For Bayesian analysis, information on the choice of priors and Markov chain Monte Carlo settings
☒	☐	For hierarchical and complex designs, identification of the appropriate level for tests and full reporting of outcomes
☒	☐	Estimates of effect sizes (e.g. Cohen's *d*, Pearson's *r*), indicating how they were calculated

Our web collection on statistics for biologists contains articles on many of the points above.

Software and code

Policy information about availability of computer code

Data collection	The raw small RNA sequencing data were obtained by custom code from BGI. The raw mRNA sequencing data were obtained from Gene Denovo Biotechnology.
Data analysis	The software SPORTS1.1 (v1.1.0) was used for small RNA sequences annotation. The HTSeq v0.6.1 was used for quantification of gene expression and gene annotations from Ensembl release. The R package DEGseq (v1.40.0) to identify the differentially expressed tsRNAs and the R package edgeR (v3.9) was employed to identify the differentially expressed genes. Microsoft Excel 2016 for layout and analysis. GraphPad Prism v7.04 and SAS 9.4 for data analysis and statistics. ImageJ bundled with 64-bit Java 1.8.0_172 for image analysis.

For manuscripts utilizing custom algorithms or software that are central to the research but not yet described in published literature, software must be made available to editors and reviewers. We strongly encourage code deposition in a community repository (e.g. GitHub). See the Nature Portfolio guidelines for submitting code & software for further information.

Data

Policy information about availability of data

All manuscripts must include a data availability statement. This statement should provide the following information, where applicable:

- Accession codes, unique identifiers, or web links for publicly available datasets
- A description of any restrictions on data availability
- For clinical datasets or third party data, please ensure that the statement adheres to our policy

The small RNA-seq and transcriptome sequencing data generated in this study have been deposited in the NCBI Sequence Read Archive (SRA) database under the BioProject accession number PRJNA687877. The data supporting this study are available in the Article, Supplementary Information, or available from the corresponding authors upon reasonable requests. Source data are provided with this paper.

Field-specific reporting

Please select the one below that is the best fit for your research. If you are not sure, read the appropriate sections before making your selection.

☒ Life sciences ☐ Behavioural & social sciences ☐ Ecological, evolutionary & environmental sciences

For a reference copy of the document with all sections, see nature.com/documents/nr-reporting-summary-flat.pdf

Life sciences study design

All studies must disclose on these points even when the disclosure is negative.

Sample size	No estimates of statistical power were performed before experiments. Animal numbers were minimized to conform to ethical guidelines while the minimal sample size was decided based on power analysis of samples of previous publications on the same project (Science. 2016 Jan 22;351(6271):397-400. Nat Cell Biol . 2018 May;20(5):535-540. Nat Commun . 2020 Mar 20;11(1):1499.).
Data exclusions	No data were excluded from the analysis.
Replication	All glucose value in Fig. 1c-e, Fig. 2f, Fig. 4b, h and Supplementary Figs. 3d, 6a, k was generated as the mean of two repeat measurements for each mouse at each time point. n numbers represent the mice used in each group, depending on the availability of mice.<br>All qRT-PCR results in Fig. 2a and Supplementary Figs. 1g, 2e-l, 3g-j, 6g-j, 7g-j were generated as the mean of two repeat measurements.<br>All ELISA results in Supplementary Fig. 2a-d were generated as the mean of two repeat measurements.<br>Four independent sets of RNA samples were used for transcriptome sequencing (Figure 1l and Supplementary Figure 1h-i). Three independent sets of sperm RNA samples were used for small RNA sequencing (Figure 3a-n, Supplementary Figure 4).<br>Northern blot in Fig. 3o and supplementary Fig. 5 are representative of three independent experiments with similar results. Western blot in Fig. 2b and Supplementary Fig. 1f, are representative of two independent experiments with similar results.<br>All replication attempts were successful.
Randomization	All mice were allocated to experimental groups on the basis of their genotype and randomized into different treatment groups.
Blinding	The genotypes of F0 mice and the LPS treatments were known to the investigators at the outset of the study. F1 mice were generated by injecting different groups of sperm RNAs into zygotes, followed by embryo transfer into surrogate mother and allocated into different cages, these processes made it impossible to be blinded to the investigators.

Reporting for specific materials, systems and methods

We require information from authors about some types of materials, experimental systems and methods used in many studies. Here, indicate whether each material, system or method listed is relevant to your study. If you are not sure if a list item applies to your research, read the appropriate section before selecting a response.

Materials & experimental systems

n/a	Involved in the study	
☐	☒	Antibodies
☒	☐	Eukaryotic cell lines
☒	☐	Palaeontology and archaeology
☐	☒	Animals and other organisms
☒	☐	Human research participants
☒	☐	Clinical data
☒	☐	Dual use research of concern

Methods

n/a	Involved in the study	
☒	☐	ChIP-seq
☒	☐	Flow cytometry
☒	☐	MRI-based neuroimaging

Antibodies

Antibodies used	Rabbit Anti-GLUT4 antibody (1:1000, BS3680, Bioworld) and rabbit Anti-Ang antibody (1:200, home-made) were used as primary antibody. Protein loading controls for each experiment used rabbit anti-GAPDH (1:10000, MB001H, Bioworld) or rabbit Anti-Na+/K+-ATPase polyclonal antibody (1:500, BS90909, Bioworld). Anti-Digoxigenin-AP Fab fragments (Roach, REF: 11093274910) used in the Northern blot experiments and is diluted in 1:10000.
Validation	The rabbit Anti-GLUT4 antibody has been validated for use in the system under study in previous publication (Curcumin regulates cell fate and metabolism by inhibiting hedgehog signaling in hepatic stellate cells. Lab Invest. 2015;95(7):790-803.).<br>The rabbit Anti-Ang antibody has been validated for use in the system under study in previous publication (Myeloid cells protect intestinal epithelial barrier integrity through the angiogenin/plexin-B2 axis. EMBO J. 2020. 39(13):e103325.).<br>The rabbit anti-GAPDH antibody has been validated for use in the system under study in previous publication (Vinexin-β protects against cardiac hypertrophy by blocking the Akt-dependent signalling pathway. Basic Res Cardiol. 2013. 108(2):338.).<br>The rabbit Anti-Na+/K+-ATPase polyclonal antibody has been validated for use in the system under study in previous publication (DHA/AA alleviates LPS-induced Kupffer cells pyroptosis via GPR120 interaction with NLRP3 to inhibit inflammasome complexes

assembly. Cell Death Dis. 2021. 12(1):73.).
The Anti-Digoxigenin-AP Fab fragments antibody from sheep is specific to digoxigenin and digoxin and shows no cross-reactivity with other steroids, such as human estrogens and androgens. This antibody is validated and extensively use in previous publications, the weblink below shows that this antibody has been used in 369 peer-reviewd papers: https://www.sigmaaldrich.cn/CN/zh/product/roche/11093274910?context=product

Animals and other organisms

Policy information about studies involving animals; ARRIVE guidelines recommended for reporting animal research

Laboratory animals	The C57BL/6 mice were maintained under a 12 h light-dark cycle in a climate-controlled (23℃, 50% humidity) specific pathogen-free condition at Laboratory Animal Center of Nanjing Agricultural University and were allocated to experimental groups on the basis of their genotype and randomized into different treatment groups. Generation and characterization of Ang-/- mice were previously described (Goncalves, K.A. et al. Angiogenin promotes hematopoietic regeneration by dichotomously regulating quiescence of stem and progenitor cells. Cell 166, 894-906 (2016).). Eight-week-old male mice were used for inflammatory model. 12-16 weeks old male mice were used for GTT, ITT, PTT and running experiment.
Wild animals	No wild animals used.
Field-collected samples	No samples collected from the field.
Ethics oversight	All studies were performed according to experimental protocols approved by the Institutional Animal Care and Use Committee (IACUC) of Nanjing Agricultural University , and all procedures were conducted in accordance with the “Guidelines on Ethical Treatment of Experimental Animals” (2006) No. 398 set by the Ministry of Science and Technology, China and the Regulation regarding the Management and Treatment of Experimental Animals” (2008) No. 45 set by the Jiangsu Provincial People’s Government.

Note that full information on the approval of the study protocol must also be provided in the manuscript.
